# How Could We Establish Monitoring and Surveillance of Health-Harming Corporations and Can Governments Be Trusted to Do It? Comment on "National Public Health Surveillance of Corporations in Key Unhealthy Commodity Industries – A Scoping Review and Framework Synthesis"

**DOI:** 10.34172/ijhpm.8621

**Published:** 2024-10-02

**Authors:** Anna B. Gilmore, Raouf Alebshehy, Stella Bialous

**Affiliations:** ^1^Tobacco Control Research Group, Department for Health, Faculty of Humanities and Social Sciences, University of Bath, Bath, UK.; ^2^Centre for 21st Century Public Health, University of Bath, Bath, UK.; ^3^School of Nursing, University of California, San Francisco, CA, USA.; ^4^Global Cancer Program, Helen Diller Family Comprehensive Cancer Center, University of California, San Francisco, CA, USA.

**Keywords:** Commercial Determinants of Health, Surveillance, Unhealthy Commodity Industry, Industry Monitoring

## Abstract

In the context of growing interest in the commercial determinants of health (CDOH) which has been defined as "the systems, practices, and pathways through which commercial actors drive health and equity," Bennett et al propose that governments implement monitoring of unhealthy commodity industries (UCIs) (including tobacco, alcohol, and ultra-processed foods) as part of their routine public health surveillance. We explore the evidence underpinning that suggestion and provide details on how corporate monitoring might be practically implemented drawing on lessons from tobacco industry monitoring which has been an established part of tobacco control. While governments should actively support such an approach as part of efforts to address commercially driven health harms, we urge caution in governments undertaking monitoring and identify significant barriers to implementation, while also suggesting ways in which those barriers might be overcome.

## Introduction

 The overwhelming evidence that some parts of the commercial sector are having an increasingly negative impact on human and planetary health has spurred interest in what is now known as the commercial determinants of health (CDOH).^1,2^ Given the scale of harm from commercial products and practices – it is estimated that just four industry products (tobacco, fossil fuels, ultra-processed food, and alcohol) account for at least a third of global annual deaths – there is an urgent need for public health action to better understand and address that harm.^1,2^

 As part of that public health action, Bennett et al propose that governments include within their routine public health surveillance systems, the monitoring of commercial practices and their impacts on health.^3^ To support this proposal, their paper seeks to develop a framework that national governments could use to monitor and mitigate commercial practices and their detrimental impacts on health. Focusing on a subset of commercial actors—corporations selling unhealthy commodities, specifically ultra-processed foods, tobacco, and alcohol—the authors undertook a scoping review of the academic literature to identify existing frameworks designed to identify or monitor corporate practices. They used content analysis to extract a list of practices detailed within such frameworks, then sought to group those practices ultimately, drawing directly on one of the frameworks identified to group practices into the five ‘environments’ that paper describes.^4^

 In the third part of their analysis, they expand their focus in two ways. First, to consider potential indicators and data sources for tracking each of the five “environments” or practices featured in the framework. Presented largely as supplementary material, this is arguably the most useful element of the work because, by identifying and providing a few examples of existing work in the area, it shows that monitoring commercial practices could be feasible. Second, they move beyond practices to flag the need to identify the specific actors that should be the focus of monitoring, noting that this needs to include the third parties and front groups that often represent corporate interests, and to extend monitoring to include the *outcomes* of commercial practices.

## Background Evidence

 The principle that we need to better understand and more effectively address commercial practices and their impacts on health is well established.^1,2^ So too is the idea that monitoring of and research on commercial actors can play a key role in this.^2,5^ The original evidence supporting such an approach comes from tobacco control where, in some settings, industry research and monitoring by academia and civil society is now well established and has played a key role in driving policy change and reducing tobacco use, in part by denormalizing the tobacco industry (Box 1). Meanwhile, growing evidence indicates that food, alcohol, gambling, and fossil fuel companies engage in the same practices,^6,7^ providing a rationale to monitor these other unhealthy commodity industries (UCIs) in the same way as tobacco. The feasibility of such an approach was established when an initial taxonomy of tobacco industry political practices^8^ formed the basis of a food industry monitoring model applied in multiple countries, again by academics.^9^

**Box 1. Tobacco Industry Research and Monitoring**

**Successes**

Research on and monitoring of the tobacco industry has played a key role in advancing tobacco control.^12^ The first such research, undertaken on whistle-blower documents was published in 1995 and, by alerting the world to its misconduct, profoundly changed attitudes to the tobacco industry.^13^ Following US Congressional hearings and litigation, it led to the release of millions more documents, the establishment of online document databases and a new area of document research in which US government funding, through its National Institutes of Health, was instrumental.^13^ The growing body of evidence helped further denormalize the tobacco industry and prompted the 2001 WHA resolution on “Transparency in Tobacco Control” which made the first formal recommendation for tobacco industry monitoring.

The early research also played a role in driving development of the WHO FCTC, negotiations for which began in 1996, and the inclusion within the Treaty of Article 5.3 which requires countries to protect their policies from the commercial and vested interests of the tobacco industry.^12^ Guidelines on Article 5.3 further established the need for tobacco industry monitoring, while other elements of the treaty required Parties to implement measures to promote public access to a wide range of information on the tobacco industry (Articles 12.c) and establish a global system that collects and disseminates information including on the activities of the tobacco industry (Article 20.4.c).^14^ Along with the earlier WHA resolution, these measures signal widespread government support for tobacco industry monitoring.

It has subsequently been observed that the countries with the most successful tobacco control policies also have active programs of industry monitoring.^15^ There has, however, been little empirical research on *how* industry monitoring or research leads to policy change. The obvious route is through tobacco industry denormalization as detailed in the early research, but case studies have documented other routes to impact.^12,16^ For example, monitoring has been used to identify and counter industry attempts to block and weaken proposed legislation and reveal how industry is circumventing legislation so loopholes can be closed.^11,12,16^

**Complexities and Failures**^17^

Although research suggests that successful implementation of the relevant treaty recommendations on industry monitoring is achievable, especially if shaped and supported by an active civil society, many countries, in all income groups, have struggled to implement effective monitoring programs.^17^ Despite government support being officially signalled via the WHA resolution and FCTC Articles detailed above, government funding for or implementation of monitoring has been limited. Most governments are also failing to ensure public access to the information on the tobacco industry recommended in the treaty.^17,18^ Instead, the most comprehensive and systematic monitoring programs have been established by academics and/or civil society actors whose monitoring and evaluation of Article 5.3’s limited implementation has also played a key role in improving accountability.^17,19^

--------------------

Abbreviations: WHA, World Health Assembly; WHO, World Health Organization; FCTC, Framework Convention on Tobacco Control.

## Learning From Tobacco Industry Monitoring

 In the context of this evidence and the urgent need to address the CDOH,^2,10^ Bennett and colleagues’ paper makes an important contribution, putting monitoring firmly on the agenda and providing a starting point for considering how to move that agenda forward. This commentary therefore attempts to build on that, by drawing on experiences – successful and otherwise (Box 1) – in implementing tobacco industry monitoring to explore how commercial practices monitoring might be implemented.^11,12^ It raises a number of interlinked issues and, above all, notes that the assumption of government support for and involvement in industry monitoring may be misplaced.

## What Monitoring Is and How It Can Most Effectively Be Used?

 Despite “monitoring” of corporations being widely referred to within public health, the term is rarely defined and interpretations and approaches have varied widely from static, intermittent observations of industry to continuous monitoring linked to action.^12^ Although Bennett et al do not define monitoring, they identify it as an area of surveillance focused specifically on corporations and, drawing on an existing definition of surveillance as “the continuous, systematic collection and interpretation of health-related data needed for the planning, implementation and evaluation of public health practice,” therefore suggest that monitoring requires a continuous and systematic approach.

 We concur with this, not least because monitoring alone is pointless: findings need to be acted upon and that requires a continuous and responsive process. Our own approach, which has a documented record of supporting policy change,^16^ integrates monitoring with investigation, research, and accountability (Figure) so that observations emerging from monitoring can, where appropriate, be rapidly investigated or subjected to more in-depth research.^11,12^ Ultimately, monitoring findings need to be actively disseminated to those who can take appropriate action and hold industry accountable. Depending on the findings, this might include civil society groups, journalists, civil servants, politicians, governments or intergovernmental organisations, lawyers, academic journals or even conference organisers.^11^ Online platforms which rapidly publish emerging evidence and informal personal networks have played a key role in this process.^12^

**Figure:**
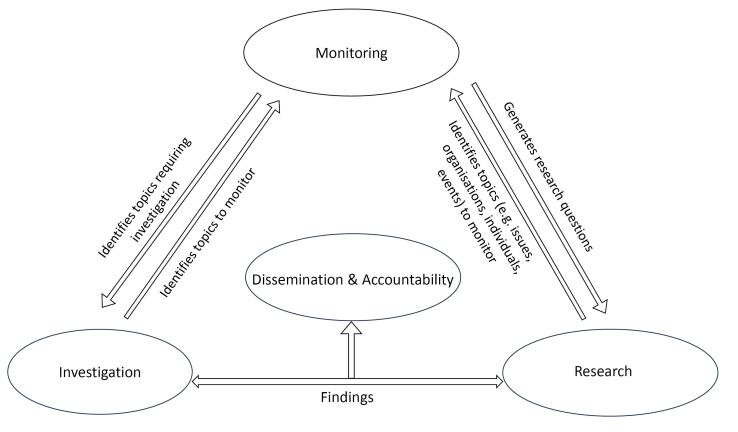
University of Bath’s Tobacco Industry Monitoring Research and Accountability Model. Note: a key element of dissemination is via the website https://tobaccotactics.org/.^11^

 There is a vast difference, however, between the collation and analysis of routine data in typical public health surveillance and the messy monitoring of large powerful corporations who often deliberately seek to hide their actions and on whom limited routine data are publicly available. Corporate monitoring is, therefore, more complex and resource intensive, politically far more sensitive and, by threatening vested interests, can pose risks to those undertaking the monitoring.^20^ Consequently, despite various requirements within the WHO FCTC for governments to implement or support tobacco industry monitoring (Box 1), many have been unable or unwilling to do so and, even where implemented, it can be hard to sustain such programs.

## Who Does the Monitoring?

 Although the authors propose that governments undertake corporate monitoring as they do routine health surveillance, they also identify government as an object of monitoring and most examples they cite—certainly those focused on monitoring corporate practices—involve civil society efforts. In relation to monitoring the “political environment,” for example, they refer to using freedom of information requests to obtain data from governments and give specific examples of Revolving Door Watch, a civil society database of European Union politicians and officials who have moved into lobbying, and the Global Tobacco Industry Interference Index, a tool developed and operationalized by civil society for monitoring tobacco industry interference in policy.^19^

 As these examples illustrate, it would be inappropriate for government to be doing that monitoring – they are often the target of the corporate influence being monitored and, in some cases, complicit in it. Even where it might be possible for governments to monitor corporate practices – in relation to the “preference shaping environment,” for example, where governments are less directly implicated – monitoring could inadvertently enable government-industry interaction which industry could exploit to exert influence. This would particularly be the case where conflicts of interest are not well understood or effectively addressed.

 In light of the above and the fact that almost all successful tobacco industry monitoring programs have involved civil society, we suggest that monitoring of commercial *practices* is more appropriately led by those outside government – non-governmental organisations, academia or both. Exceptions include settings where there is no civil society or where monitoring of government-owned tobacco companies poses specific risks to them. In these instances, monitoring by committed civil servants, typically within ministries of health, has proved vital.

 Governments can, however, play a more direct role in monitoring *outcomes* which, as the authors note include consumption patterns, incidence and prevalence of disease, topics often already included in health surveillance.^3^ We suggest, however, that further work is needed to extend these outcomes to make them genuinely useful in corporate monitoring: first to link outcomes to corporate products and practices; second, to estimate the costs of these outcomes (or harms) on society; third to begin to attribute these harms and costs to specific industries and corporations. Such data will be essential if we are to move towards using full cost accounting (or “polluter pays”) approaches to addressing commercial harm.^2^ They also have the advantage of ensuring governments become increasingly aware of the scale and cost of commercial harm, a potential stimulus for action.

## Next Steps

 It is clear from the above that, even in the case of tobacco where it is *required*, corporate monitoring has faced considerable political, resource and other practical constraints, succeeding only in limited settings. Alongside tobacco industry research it has, nevertheless, played an essential role in advancing tobacco control (Box 1). This disproportionate impact likely reflects the global nature of the industry involved, such that findings in one jurisdiction have relevance well beyond that jurisdiction, including regionally and globally. Initially, therefore, wider UCI monitoring is likely to be established opportunistically by those able to overcome those constraints, who, given the global nature of other UCIs, should be encouraged to consider this global role in terms of information gathering, sharing and impact.

 Although we urge caution in governments alone undertaking corporate monitoring (other than when focused on *outcomes *or in specific settings, as detailed above), governments do have a duty to protect health and should, therefore, support monitoring. Such support can come in various forms: implementing a formal legislative requirement for monitoring, protecting those undertaking monitoring, providing funding, and statutorily requiring corporations to report on their practices including, for example, marketing and lobbying expenditures and product pricing.

 In reality, achieving this support will be difficult. Within tobacco control, governmental and intergovernmental action followed initial revelations of the industry’s misconduct (Box 1). Consequently, early monitoring programs can, by contributing to such revelations and subsequent industry denormalization, help secure the political leadership and support required for more formal and widespread implementation.

 Within such efforts, Bennett and colleagues’ framework could be tested as an option for corporate surveillance. It can be updated with emerging literature including detailed taxonomies of specific UCI practices^6,7^ and datasets relevant to the CDOH. The proposed focus could also be expanded beyond ultra-processed foods, tobacco, and alcohol to other UCIs, notably fossil fuel and gambling industries, given the similarity in their documented practices, including a shared “playbook” of political and scientific practices.^1,6,7^ This includes operating through the same think tanks, front groups and public relations companies, for example. A coordinated approach to monitoring across industries and geographies, while difficult to achieve, could therefore enable significant economies of scale to be realised. Much could initially be achieved by harnessing existing online platforms to create single or linked profiles of such organisations. Longer-term, a focus beyond UCIs would require the inclusion of other corporate practices that can harm health often regardless of the product the corporation sells—supply chain and labour practices,^1,12^ for example.

 Ultimately, a polluter pays approach involving statutory levies on industries could be used to fund corporate monitoring and research, building on models in Italy, California, and Thailand, where levies on pharmaceutical, tobacco, and alcohol industries have been used to fund independent research on their products.^6^ Such approaches require appropriate safeguards to ensure that the industries in question are not able to misrepresent these levies as voluntary donations and leverage them to secure influence. Efforts to move the broader agenda on the CDOH forward, including the establishment of a new WHO work program on this topic, are under development and will be essential to enabling progress towards this point.^2,10^

## Ethical issues

 Not applicable.

## Conflicts of interest

 Authors declare that they have no conflicts of interest.
